# Biological hierarchy from genotype to phenotype in syndromic and non-syndromic autism spectrum disorder

**DOI:** 10.3389/fnins.2026.1964684

**Published:** 2026-09-25

**Authors:** Zemin Qing, Ziyan Yin, Bin Chen, Zuyao Rao, Dandan Yin, Dehai Li, Yuxuan Lu, Mengmeng Shi, Shen Li, Yanghuan Xu, Kai Cheng, Wenhua An, Can Yuan, Qiong Dai

**Affiliations:** 1 Department of Health, Maternal and Child Health Hospital of Hubei Province, Tongji Medical College, Huazhong University of Science and Technology, Wuhan, China; 2 Department of Maternal, Child and Adolescent Health, School of Public Health, Tongji Medical College, Huazhong University of Science and Technology, Wuhan, China; 3 Department of Pharmacy, Wuhan Mental Health Center, Wuhan Psychological Hospital, Tongji Medical College, Huazhong University of Science and Technology, Wuhan, China; 4 The Second Clinical Medical College, Guangdong Medical University, Dongguan, China

**Keywords:** autism spectrum disorder, chromatin remodeling, epigenetic regulation, hierarchical framework, non-syndromic ASD, synaptic dysfunction, syndromic ASD

## Abstract

Autism spectrum disorder (ASD) is a heterogeneous neurodevelopmental condition whose pathophysiology is thought to arise from complex interactions among genetic variation, molecular and cellular perturbations, and neural circuit dysfunction. Although the distinction between syndromic and non-syndromic ASD was introduced as a pragmatic clinical classification, these clinically defined subgroups may exhibit overlapping yet distinct patterns of genetic liability and molecular dysregulation while also exhibiting mechanistic convergence. This narrative review examines the molecular mechanisms underlying synaptic development, structure, and function, with particular emphasis on the roles of chromatin remodeling and epigenetic regulation in neurodevelopment. It further considers how molecular and cellular alterations may contribute to impaired functional integration at the neural circuit level. By comparing the shared and unique genes and pathways associated with syndromic and non-syndromic ASD, the review identifies potential points of mechanistic convergence across multiple levels of biological organization. To integrate these findings, the review proposes a three-layer hierarchical framework that links gene function (Layer 1), core biological pathways (Layer 2), and clinical phenotypes (Layer 3). By mapping ASD-associated genes onto a functional continuum ranging from local synaptic effectors to global regulators of chromatin and gene expression, this framework illustrates how subtype-enriched genetic factors may give rise to both shared and unique molecular, cellular, and circuit-level alterations. The proposed framework may help identify common and subtype-enriched biological mechanisms, inform biomarker discovery, and provide a conceptual basis for mechanism-informed ASD research and biologically grounded clinical stratification.

## 1 Introduction

Autism spectrum disorder (ASD) is a neurodevelopmental condition characterized by persistent challenges in social communication and interaction, accompanied by restricted and repetitive patterns of behavior and interests (Hirota and King, 2023). This diversity in presentation suggests that ASD does not constitute a single disease entity; it represents a shared behavioral outcome arising from multiple, partially overlapping biological pathways (Paulsen et al., 2022). Genetic research reveals that ASD is highly heritable (Tick et al., 2016). Large-scale genomic studies show that many high-impact variants are pleiotropic, conferring risk for ASD as well as intellectual disability (ID), epilepsy, and other neurodevelopmental or psychiatric conditions (Al-Sarraj et al., 2021). These findings highlight the intrinsic genetic heterogeneity of ASD and the need to clarify its biological basis to refine pathophysiological models (Litman et al., 2025). However, cataloging ASD risk genes alone cannot explain how diverse genetic perturbations converge on shared ASD-related phenotypes. A hierarchical framework is needed to organize these genes, link their functions to pathway abnormalities and clinical features, and distinguish common mechanisms from those enriched in specific ASD subgroups.

Although the syndromic versus non-syndromic distinction originated as an operational clinical classification rather than a strict biological dichotomy, accumulating molecular evidence suggests that these clinically defined subgroups may exhibit partially distinct patterns of genetic liability and molecular perturbation (Rudhra and Singh, 2026). This distinction may serve as a heuristic framework for investigating the biological heterogeneity and convergent pathogenic mechanisms of ASD. Non-syndromic ASD is characterized by the absence of consistent syndromic diagnoses or systemic anomalies (Kereszturi, 2024). In contrast, syndromic ASD is associated with identifiable genetic or genomic syndromes, often accompanied by ID as well as somatic or neurological abnormalities (Bozhilova et al., 2023). Recent reviews have examined associations between ASD risk genes and clinical phenotypes (DiStefano et al., 2025), but the points of convergence and divergence between non-syndromic ASD and syndromic ASD across key risk genes, core signaling pathways, and subsequent molecular and cellular consequences remain incompletely defined. Few studies have systematically mapped the genetic architecture onto a hierarchical regulator-effector framework that distinguishes regulators of chromatin and transcription from effectors involved in synaptic and ion-channel activity, or examined how dose-sensitive bridge genes such as *SHANK3*, *FMR1*, and *UBE3A* span these categories.

This narrative review integrates evidence from human genetics, functional genomics, experimental models, and mechanistic studies to examine how ASD-associated genetic variation contributes to molecular pathways, cellular dysfunction, neural circuit alterations, and clinical phenotypes. To establish a biologically informed framework, representative genes were derived from a data-informed selection strategy based on the Simons Foundation Autism Research Initiative (SFARI) Gene database.^1^ Genes with strong genetic evidence (SFARI Gene Score 1 or Score 2) were considered as the primary gene pool and further categorized according to syndromic and non-syndromic annotations. Genes with relatively strong evidence scores (EAGLE) and substantial literature support were prioritized for targeted mechanistic literature retrieval. Biological pathways discussed in this review were informed by pathway enrichment analyses of the selected ASD-associated gene set and interpreted in the context of published molecular, cellular, developmental, and clinical evidence.

The selected genes and pathways are intended to represent mechanistically informative examples rather than an exhaustive catalogue of ASD-associated genes or biological processes. This framework aims to facilitate interpretation of genotype-to-phenotype relationships across syndromic and non-syndromic ASD while acknowledging the substantial biological overlap between these categories.

## 2 Molecular mechanisms

### 2.1 Key genes for non-syndromic ASD

Current evidence indicates that risk genes for non-syndromic ASD are enriched in molecular pathways that regulate neuronal synaptic function and electrophysiological activity, thereby mediating core pathological processes from genetic susceptibility to aberrant neural circuit operation.

#### 2.1.1 Synaptic structure and function related genes

These genes encode core molecular components essential for synapse assembly and function, directly impacting inter-neuronal communication.

Postsynaptic Scaffold and Adhesion Molecules: Within the postsynaptic density (PSD) of excitatory synapses, *SHANK2* and *SHANK3* encode scaffold proteins. These proteins organize glutamatergic synapses by binding to other scaffold molecules such as DLG4 and Homer proteins, as well as N-methyl-D-aspartate (NMDA) (Lee et al., 2022) and α-amino-3-hydroxy-5-methyl-4-isoxazolepropionic acid (AMPA) receptors (Jia et al., 2025). Disruption of SHANK family proteins alters PSD architecture, receptor localization, and subsequent signaling (Bucher et al., 2021), leading to impaired synaptic transmission and plasticity that recapitulates autism-related phenotypes in animal models (Eltokhi et al., 2021; Jung and Park, 2022). Neuroligins encoded by *NLGN2*, *NLGN3*, and *NLGN4X* reside at the postsynaptic membrane and form trans-synaptic adhesion complexes through high-affinity interactions with presynaptic neurexins encoded by *NRXN1*, *NRXN2*, and *NRXN3*. These complexes regulate synapse initiation, differentiation into excitatory or inhibitory subtypes, and the organization of neurotransmitter release sites. Mutations in neuroligins and neurexins have been repeatedly linked to non-syndromic ASD and related neurodevelopmental conditions (Yoshida et al., 2021).

Glutamate Receptor Genes: *GRIN1*, *GRIN2A*, and *GRIN2B* encode subunits of the NMDA receptor, a calcium-permeable receptor essential for long-term potentiation (LTP) and other forms of synaptic plasticity that underlie learning and memory. *GRIA2* encodes a subunit of the AMPA receptor that dominates rapid excitatory postsynaptic currents and controls the moment-to-moment gain of glutamatergic synapses. Rare, often *de novo*, missense or truncating variants in these genes alter receptor kinetics, calcium signaling, and synaptic strength, and have been implicated in ASD (Deutsch et al., 2022; Sheng et al., 2022; Gjerulfsen et al., 2024).

#### 2.1.2 Ion channel and neuronal excitability modulating genes

These genes critically regulate neuronal membrane properties and action potential generation, directly influencing neural circuit excitability and the E/I (excitation/inhibition) balance.

Voltage-Gated Ion Channels: *SCN2A* and *SCN8A* encode alpha subunits of voltage-gated sodium channels concentrated at the axon initial segment and along the axon. These channels are crucial for the initiation and propagation of action potentials and for sustaining high-frequency firing. Both gain-of-function and loss-of-function (LoF) variants in *SCN2A* are associated with ASD, indicating that either excessive or insufficient excitability can perturb circuit development and function (Scott et al., 2025). *KCNQ2* and *KCNQ3* encode subunits of potassium channels that generate the M current, a slowly activating outward current that stabilizes the resting membrane potential and modulates neuronal adaptation to sustained depolarization. Variants in these genes alter intrinsic excitability and predispose neurons to network-level instability (Ma et al., 2023; Oh et al., 2023).

Inhibitory Receptors: *GABRB2* and *GABRB3* encode beta subunits of the gamma-aminobutyric acid (GABA) type A receptor (Menzikov et al., 2021), which mediate fast inhibitory chloride currents. Altered function of these receptors disrupts the functional integration of pyramidal neuron subtypes in cortical circuits (Babij et al., 2023) and has been linked to an imbalance between excitation and inhibition in cortical and subcortical circuits, a widely proposed mechanism in ASD pathophysiology (Babij et al., 2023; Coskunpinar et al., 2023).

#### 2.1.3 Cytoskeletal, signal transduction, and transcription regulatory genes

Cytoskeletal Anchoring: *ANK2* and *ANK3* encode ankyrin proteins. These molecules anchor voltage-gated sodium channels and cell adhesion molecules to specific membrane domains (e.g., axon initial segment, nodes of Ranvier), regulating neuronal polarity, electrophysiological domain function, and action potential threshold (Ding et al., 2024; Nelson et al., 2024).

Transcription Factors and Signaling Proteins: *TBR1* is a transcription factor that specifies the identity and projections of corticothalamic and other deep-layer neurons and is critical for the development of cortical regions implicated in higher cognition and language (Crespo et al., 2022). *RELN* encodes the secreted protein Reelin, which guides neuronal migration and cortical lamination, and thereby influences the assembly of cortical macro- and microcircuitry (Di Donato et al., 2022; Riva et al., 2024).

#### 2.1.4 Other functionally classified genes

RNA Processing and Local Translation: *CELF4* encodes an RNA-binding protein regulating the splicing, stability, and local translation of mRNAs encoding synaptic proteins. This influences synaptic proteome composition and excitatory network homeostasis (Salamon et al., 2023).

Presynaptic Vesicle Release: *RIMS1* (also known as *RIM1*) encodes a core presynaptic active zone protein. It organizes calcium channels and vesicle docking machinery, precisely controlling vesicle release probability and short-term synaptic plasticity (Muller et al., 2022).

Functional disturbances in this domain can induce local and systemic network dysfunction even in the absence of large-scale structural abnormalities, making it a critical component of the biological basis of non-syndromic ASD (Pilorge et al., 2016). Recent work integrating genome-wide association study (GWAS) results with transcriptomic and proteomic data suggests that common risk variants are enriched in regulatory regions of genes expressed in developing cortical neurons, particularly those involved in synaptic transmission, ion channel activity, and chromatin remodeling (Wingo et al., 2025). The polygenic risk for ASD appears to modulate the same synaptic and electrophysiological pathways as rare disruptive variants, albeit with much smaller effect sizes.

Whereas non-syndromic ASD is predominantly driven by synaptic effector genes, syndromic ASD shows enrichment for variants affecting higher-order regulatory nodes that govern chromatin organization and global cellular signaling.

### 2.2 Key genes in syndromic ASD

Studies reveal that pathogenic variants in syndromic ASD are highly enriched in two broad classes: regulatory node genes governing development and primary transcription, and core functional nodes at critical synapses and signaling pathway intersections. Together, these variants form a multi-level pathological network spanning from the chromatin layer to the cellular and circuit levels.

#### 2.2.1 Epigenetic regulation and chromatin remodeling-related genes

This gene category comprises regulators of gene expression that modify chromatin states, three-dimensional genome organization, or transcriptional processes. In syndromic ASD, disruption of these epigenetic mechanisms can globally perturb developmental gene expression, leading to widespread neural and systemic abnormalities.

Genes such as *SETD5*, *KMT2A*, *CREBBP*, *EP300*, *CHD7*, *SIN3A*, and *ZMYND8* significantly influence the spatiotemporal expression of neurodevelopmental and synapse-related genes (Dias et al., 2022; Ahsan et al., 2023). They achieve this by modifying histone marks and chromatin structure, particularly topological associating domains (TADs). TADs are crucial for organizing transcriptional networks during neurodevelopment (Okhovat et al., 2023). Dysfunction of these genes can lead to systemic disruptions in developmental programming and plasticity regulation (Ogino and Dou, 2024; Williams et al., 2024). Previous work on the epigenome in neurodevelopmental disorders has been systematically reviewed, highlighting the central role of chromatin remodeling in shaping neurodevelopmental trajectories (Reichard and Zimmer-Bensch, 2021). However, many of these insights derive from cellular models or bulk tissue analyses in animals, which may not fully capture the cell type specificity and spatiotemporal complexity of chromatin dynamics in human cortical development. Consequently, the specific pathogenic pathways secondary to TAD disruption likely vary across different forms of syndromic ASD and require further validation in human-relevant systems.

*NIPBL* serves as a cohesin loading factor involved in chromatin looping and TAD formation, while *HNRNPU* functions as a nuclear matrix protein maintaining spatial organization between chromatin and the nuclear scaffold. Variations in these genes can trigger large-scale transcriptional network rearrangements, resulting in multisystem developmental abnormalities and brain structural alterations (Oksanen et al., 2026).

*MEF2C*, *BCL11A*, and *RAI1* have been implicated in neurodevelopmental and behavioral regulation (Comajuan et al., 2026; Rosoff et al., 2026), while *ADNP* functions in transcriptional and chromatin regulation (D'Incal et al., 2026). Mutations in these genes can disrupt cortical and subcortical network architecture and function, often associated with syndromic ASD (Bennison et al., 2023).

#### 2.2.2 Signaling pathways and genes regulating cell growth

Mutations in key signaling pathway genes that regulate cell proliferation, differentiation, and metabolism are critical drivers of syndromic ASD. LoF variants in these genes are often associated with early-onset epilepsy, severe developmental delay, and pronounced ASD phenotypes. *TSC1* and *MTOR* are integral to the mammalian target of rapamycin (mTOR) pathway (Gerasimenko et al., 2023), *PTEN* negatively modulates the phosphatidylinositol 3-kinase (PI3K)/protein kinase B (AKT)/mTOR pathway (Glaviano et al., 2023), *NF1* and *BRAF* regulate Ras/mitogen-activated protein kinase (MAPK) signaling as Ras-GAP and MAPK cascade kinases, respectively (Wang J. et al., 2025), while *DYRK1A* impacts brain volume and cortical structure by influencing the cell cycle and neuronal differentiation (Morrow, 2021). These mutations contribute to mesolimbic, cerebellar, and cortical dysplasia, hamartomas, tumor susceptibility, and a high prevalence of ASD (Nguyen et al., 2024).

#### 2.2.3 Cross-level bridge genes

This category comprises genes that directly regulate specific biological processes, including synaptic function, protein homeostasis, cytoskeletal organization, and RNA metabolism. Although these genes act as molecular effectors, their expression and function are often shaped by chromatin and epigenetic regulators, global signaling pathways, and cellular growth control networks, positioning them as effector nodes within the multilevel architecture of syndromic ASD. Within this architecture, functionally diverse genetic perturbations can converge on overlapping neurodevelopmental phenotypes.

*SHANK3* and *SYNGAP1* are key postsynaptic proteins, with *SHANK3* serving as the core scaffold of the PSD and SYNGAP1 functioning as a postsynaptic Ras-GAP. *SHANK3* mutations, particularly deletions or loss-of-function variants, are associated with Phelan-McDermid syndrome (Jia et al., 2025). *STXBP1* is essential for presynaptic vesicle release (Long et al., 2025), *SLC6A1* encodes the GABA transporter (Shah et al., 2025), and *KCNB1* encodes the Kv2.1 potassium channel (Manville et al., 2024).

*UBE3A* encodes an E3 ubiquitin ligase predominantly expressed from the maternal allele in neurons and serves as the core pathogenic gene of Angelman syndrome (Gupta et al., 2025). *USP9X*, a deubiquitinase, removes ubiquitin chains from target proteins, preventing their proteasomal degradation and regulating their activity and localization (Wang et al., 2022). *UBE3A* and *USP9X* operate at opposing yet complementary points in the ubiquitination and deubiquitination pathways. Mutations in these genes disrupt protein homeostasis by altering substrate protein half-life, abundance, and functional state (Avagliano Trezza et al., 2021). Given the reliance of neurons and synapses on precise protein turnover and local protein synthesis, such imbalances can result in abnormalities in synaptic number, structure, and plasticity, thereby leading to cognitive deficits, seizures, and autism-related behavioral phenotypes.

*FMR1* encodes the fragile X messenger ribonucleoprotein (FMRP), an RNA-binding protein that binds to multiple mRNAs. Its primary function is to inhibit or finely regulate the local translation of these mRNAs at synapses (Hilal et al., 2025). *VPS13B* is a large Golgi-associated protein that plays a crucial regulatory role in vesicle transport and organelle structure maintenance. Mutations in *VPS13B* can lead to developmental and degenerative changes in the nervous system and tissues such as the retina, representing a typical mechanism of multisystem syndromic ASD (Ugur et al., 2024). *MAP1A* is a microtubule-associated protein widely expressed in mature neurons, involved in microtubule stabilization, axonal and dendritic structure maintenance, and intracellular transport. Pathogenic variants of *MAP1A* are often associated with widespread neurodevelopmental disorders and autism-related behavioral phenotypes (Demontis et al., 2026). *MEIS2* is a homeobox transcription factor involved in developmental regulatory networks and organogenesis (Delgado et al., 2021). It has also been implicated in autism-associated neurodevelopmental and peripheral phenotypes (Roussel et al., 2022). *CAMK2A* encodes the calcium/calmodulin-dependent protein kinase II alpha subtype, which plays important roles in synaptic plasticity, learning, and memory and has been implicated in neurodevelopmental disorders with intellectual disability and autism-related features (Lintas et al., 2023).

*FMR1*, *VPS13B*, *MAP1A*, *MEIS2*, *IQSEC2*, and *CAMK2A* constitute a functionally interconnected pathogenic cascade. Together, they extend from transcriptional regulation to synaptic execution, encompassing multiple levels, including synaptic local translation (Wilkerson et al., 2023), Golgi apparatus and vesicle transport (Zorn et al., 2022), microtubule and dendritic morphology, developmental transcription programs, postsynaptic small GTPase signaling, receptor trafficking (Delgado et al., 2021), and Ca^2+^-dependent synaptic plasticity (Lintas et al., 2023). Functional imbalances in this pathway often lead to core symptoms of ASD, accompanied by ID, epilepsy, and multisystem abnormalities, reflecting the multilevel biological perturbations associated with syndromic ASD.

Broad-acting neurodevelopmental regulator genes exert broad influences on chromatin states, transcriptional programs, and cellular signaling. Patients carrying distinct mutations in these genes consistently display core behavioral characteristics of ASD (Tabbaa et al., 2023), though the underlying biological mechanisms remain incompletely understood. Emerging evidence suggests that molecular redundancy within chromatin remodeling complexes and homeostatic feedback loops may partially buffer against single-gene loss and dampen the spread of dysregulation (Rozenfeld et al., 2023; Fink et al., 2024). A key question is whether functionally distinct regulator genes converge upon shared circuit-level mechanisms. Recent studies of ASD mouse models point to a shared disturbance at the level of hippocampal circuits, which may provide a mechanistic convergence for diverse genetic risks (Keith et al., 2025). Across models, alterations in the balance between excitation and inhibition are a consistent feature. *FMR1* knockout mice show hypersynchronous population activity, larger and more stable neuronal assemblies, and a shift toward excessive excitation in hippocampal networks (Bataveljic et al., 2024). In contrast, mice deficient in *Mecp2* (a model of Rett syndrome) display weaker assemblies, reduced pyramidal cell firing, and a bias toward inhibition (Xu et al., 2022). These opposing changes converge on abnormal oscillatory dynamics, including altered gamma and theta rhythms, which are critical for hippocampal functions such as cognitive mapping and social memory (Janz et al., 2025). Glial mechanisms further contribute to this circuit-level instability. Astrocytes in *FMR1* knockout mice show reduced branch complexity and altered calcium signaling, which can disturb glutamate uptake and neuromodulator handling, thereby amplifying excitatory drive (Bataveljic et al., 2024). Microglial changes, including abnormal synaptic pruning and altered surveillance, have been reported in several ASD-related models and may disrupt the normal refinement of hippocampal connectivity (Saitoh et al., 2021). Thus, impaired hippocampal network stability and coordination may underlie shared cognitive and social deficits in ASD (Bhamidimarri et al., 2026).

### 2.3 Insights from overlapping genes

*SHANK3* is implicated in both syndromic and non-syndromic ASD, exemplifying how a single locus can span hierarchical levels of ASD pathology. The gene resides on the medial postsynaptic membrane of excitatory glutamatergic synapses and encodes a core scaffold protein within the PSD (Jia et al., 2025). Through its PDZ and proline-rich domains, *SHANK3* organizes large multiprotein complexes that include PSD-95, SAPAP, Homer, and both metabotropic and ionotropic glutamate receptors. This scaffold architecture supports the structural integrity of dendritic spines and the geometric organization of the PSD (Chen et al., 2023). This architecture anchors glutamate receptors at the postsynaptic membrane and couples subsequent signaling pathways, including Ras-MAPK and mTOR cascades, essential for LTP, depression, and other synaptic plasticity processes (Barone et al., 2026).

Disruptive *SHANK3* variants, such as haploinsufficiency and deletions, are linked to reduced excitatory synapse numbers, altered spine morphology, and impaired excitatory synaptic transmission. These changes are believed to diminish the efficiency of information processing in social and cognitive networks, particularly in prefrontal and temporoparietal association regions (Yang et al., 2025). In non-syndromic ASD, *SHANK3* mutations primarily manifest as direct disruptions to synaptic architecture and function without consistent extra-neurological anomalies (Hamoudi et al., 2022; Belenska-Todorova et al., 2025). Conversely, *SHANK3* haploinsufficiency underpins Phelan-McDermid syndrome, characterized by ASD alongside severe language deficits, neonatal hypotonia, and other developmental abnormalities (Pagano et al., 2026). While synaptic dysfunction underlies the core ASD phenotype, *SHANK3* may also influence broader aspects of early brain and systemic development, with the magnitude and timing of its disruption potentially exerting widespread developmental effects (Nakamura et al., 2026). Recent neuroimaging evidence suggests that *SHANK3* deficiency produces distinct patterns of cortical surface morphology disruption compared to non-*SHANK3* ASD (Li et al., 2021). This finding is consistent with a dosage sensitivity threshold model in which phenotypic severity depends not only on gene identity but also on the magnitude and developmental timing of genetic perturbation. When *SHANK3* function remains above a critical threshold, its effects may be largely confined to the synaptic effector level. In contrast, more severe dosage reduction may compromise cellular homeostasis and developmental timing, thereby engaging higher-order regulatory pathways and broadening the phenotype from predominantly synaptic ASD to syndromic ASD. This model provides a mechanistic basis for understanding how variants affecting the same locus can engage different levels of the pathogenic hierarchy and produce distinct clinical presentations.

Full mutation of *FMR1*, with large cytosine-guanine-guanine repeat expansions that silence the gene, is strongly associated with fragile X syndrome and a high rate of ASD (Winarni et al., 2025). At the same time, premutation carriers, who have intermediate repeat lengths, typically do not meet criteria for classic fragile X syndrome, yet they show increased rates of non-syndromic ASD (Lau et al., 2025). These observations indicate that partial disruption of FMRP function or RNA-mediated toxicity can alter neural circuits sufficiently to produce core autistic traits without triggering the full syndromic phenotype (Hilal et al., 2025).

Loss of *UBE3A* in neurons leads to impaired ubiquitin-dependent protein degradation and widespread dysregulation of substrates that are important for synaptic function and neuronal excitability, consistent with a multisystem model of syndromic ASD (Pandya et al., 2021). In contrast, duplications and triplications of the chromosomal region 15q11-q13, which increase *UBE3A* dosage, are strongly associated with non-syndromic ASD and epilepsy (Fink et al., 2021). Overexpression of *UBE3A* appears to disturb synaptic homeostasis through excessive ubiquitin pathway activity, yet often spares the severe somatic anomalies that characterize Angelman syndrome (Nenninger et al., 2022). The phenotypic outcome therefore shows a strong dosage dependence. Haploinsufficiency is typically associated with a severe syndromic disorder, whereas increased dosage can predispose to non-syndromic forms of ASD.

Non-syndromic ASD is frequently described as involving synaptopathic and channelopathic mechanisms. By contrast, syndromic ASD is more often associated with variants affecting chromatin remodeling, transcriptional regulation, intracellular signaling, and early neuronal development, although substantial mechanistic overlap exists between these categories.

Comparison of genes, pathogenic processes, and mechanisms between non-syndromic and syndromic ASD is presented in Table 1.

**Table 1:** Comparison of genes, pathogenic processes, and mechanisms between non-syndromic and syndromic ASD.

Dimension for comparison	Non-syndromic ASD	Syndromic ASD	Key implications
Core functional tier and representative genes	Synaptic structure and function layer: Synaptic scaffold and adhesion (e.g., *SHANK3*, *NLGN3*/*4X*). Ion channels (e.g., *SCN2A*, *KCNQ2*). Neurotransmitter receptors (e.g., *GRIN2B*, *GABRB3*).	Chromatin and transcriptional regulatory layer: Chromatin remodeling and epigenetic regulators (e.g., *CHD7*, *SETD5*). Global signaling nodes (e.g., *TSC1*, *PTEN*, *NF1*). Transcription regulators (e.g., *MEF2C*, *ADNP*).	Locates the primary level of initial pathological disruption. Reflects fundamental differences in genetic etiology.
Genetic model	Often polygenic/oligogenic contributions; rare high-impact variants can also contribute	Often monogenic or CNV-defined etiologies, although genetically heterogeneous.	Reflects commonly observed differences in genetic architecture rather than absolute categories.
Primary molecular function	Mediates synapse-specific signal transmission and electrical activity; maintains neuronal E/I balance.	Regulates genome-wide transcriptional programs, cell cycle, metabolism and growth; influences broad cell fate decisions.	Synaptic Layer genes: concrete, localized functions. Chromatin Layer genes: broad, regulatory functions.
Core mechanism paradigm	Synaptopathy: directly disrupts synaptic structure, plasticity, and neurotransmission, leading to microcircuit dysfunction.	Dysregulated developmental programs: disrupts gene expression networks, causing multisystem developmental anomalies.	Explains phenotypic specificity: synaptic connectivity vs. global neurodevelopmental program.
Pathogenic initiation site	The functional unit of a synapse or neuronal membrane.	Chromatin, transcriptional network, or global cellular signaling pathways.	Maps the origin of pathogenesis from molecular to cellular level.
Typical cellular phenotypes	Abnormal dendritic spine morphology, defective receptor trafficking, erroneous PSD assembly, altered neuronal excitability.	Aberrant cell proliferation/differentiation/migration, altered cell size and structure, disrupted cortical laminar organization, neoplastic lesions.	Reflects a spectrum from micro-connectivity defects to macro-structural malformations.
Impact on neural circuits	Relatively localized, primarily affecting information processing efficiency and plasticity of specific neural circuits.	Broad and profound, affecting overall architectural assembly, regional connectivity, and systemic function of neural circuits.	Connects molecular defects to macro-level brain functional abnormalities.
Phenotypic breadth	Relatively limited, predominantly neurological/behavioral symptoms.	Widespread, multisystem involvement with typical somatic features.	Defines the clinical boundary between the two ASD subtypes.
Associated clinical features	Core ASD symptoms are prominent; ID, epilepsy, and other comorbidities may occur with variable frequency.	ID, epilepsy, and multisystem abnormalities are common in many syndromic conditions, but severity and specific features vary.	Clinical features show substantial variability and overlap between categories.
Potential therapeutic strategies	Pharmacological correction targeting specific receptors/channels (e.g., NMDA/AMPA receptor positive allosteric modulators).	Pathway inhibitors (e.g., mTOR inhibitors), epigenetic regulators, gene replacement therapies.	Highlights potentially different mechanistically informed therapeutic approaches.
Illustrative case (SHANK3)	Mild LoF variants: mainly impair synaptic function, typically presenting as non-syndromic ASD.	Severe LoF (e.g., deletions): cause Phelan-McDermid syndrome, featuring global developmental delay, hypotonia, typical facies, etc.	Within the same gene, perturbation severity and molecular mechanism may influence the resulting phenotype.

ASD, autism spectrum disorder; AMPA, α-amino-3-hydroxy-5-methyl-4-isoxazolepropionic acid; ID, intellectual disability; LoF, loss of function; mTOR, mammalian target of rapamycin; NMDA, N-methyl-D-aspartate; PSD, postsynaptic density.

## 3 The key signaling pathways

### 3.1 Key signaling pathways for non-syndromic ASD

#### 3.1.1 Neural development and synaptic function pathways

The immunoglobulin superfamily cell adhesion molecule pathway exemplifies the principle of highly specific cellular recognition and connectivity. These pathways, encompassing trans-synaptic adhesion molecules like neurexins and neuroligins (Li et al., 2026) and core glutamatergic signaling components (Francis et al., 2025), are known to be essential for precise synapse formation, stabilization, and excitatory neurotransmission (De Luca et al., 2025), with their disruption contributing directly to synaptic dysfunction and altered neural circuit connectivity in ASD. Disruption of these pathways in non-syndromic ASD primarily manifests as subtle but functionally relevant alterations in connectivity patterns, excitatory transmission, and network stability, rather than major structural abnormalities (Sindi, 2023). This indicates that variations within specific synaptic recognition and transmission systems are sufficient to elicit the core behavioral phenotypes of ASD. Analyzing these pathways through a hierarchical framework spanning gene function, pathway activity, and clinical phenotype allows for the elucidation of their ASD-specific effects beyond broader neurodevelopmental roles. This clarifies how perturbations in these shared synaptic mechanisms predominantly contribute to a non-syndromic ASD presentation.

#### 3.1.2 Synaptic plasticity-related pathways

Dysregulation of the LTP cascade (Caya-Bissonnette and Beique, 2024), encompassing NMDA receptor-mediated calcium influx, subsequent kinase activity, and AMPA receptor trafficking (de Leon-Lopez et al., 2025), is implicated in impaired synaptic plasticity and experience-dependent circuit remodeling in ASD (Payeur et al., 2021). Ras and Rap1 GTPase pathways integrate higher-order signals that regulate LTP induction and maintenance, making them potential points of vulnerability for neural circuit function (Lopez-Garcia et al., 2024; Lopez-Merino et al., 2025). In non-syndromic ASD, genetic perturbations may be relatively enriched in synapse-proximal and activity-dependent mechanisms, including Ras and Rap1 signaling, thereby affecting AMPA receptor trafficking, synaptic retention, and activity-dependent circuit adaptation. It is essential to distinguish between pathway mechanisms shared across ASD and those enriched in syndromic or non-syndromic subgroups by examining synaptic plasticity pathways in relation to ASD-associated gene classes and clinical phenotypes.

#### 3.1.3 Signal transduction pathways

Intracellular signal transduction pathways connect extracellular signals to localized synaptic responses, with the Ras signaling pathway playing a crucial role in regulating essential neurodevelopmental processes and mediating synaptic activity through localized changes in receptor trafficking (Bahar et al., 2023; Bjornson et al., 2023), cytoskeletal organization (Rothenberg et al., 2023), and synaptic plasticity (Kim et al., 2024). Their recurrent involvement in non-syndromic ASD suggests an increased vulnerability within this synaptic effector layer. These pathways integrate developmental and neuromodulatory signals with activity-dependent gene expression, neuronal morphology, and synaptic maintenance. Their relevance to non-syndromic ASD is underscored by the preferential involvement of synaptic signal transduction, receptor trafficking, and dendritic spine regulation. Thus, in non-syndromic ASD, genetic risk converges on mechanisms governing localized synaptic signaling and microcircuit refinement.

#### 3.1.4 Reward, motivation, and predictive processing pathways

The addiction pathway reflects the functional organization of the mesolimbic and mesocortical circuits, which link midbrain dopaminergic nuclei with the nucleus accumbens and prefrontal cortex and involve interactions between glutamatergic and GABAergic systems (Ebrahimi et al., 2024). Dysfunction within these reward and motivation networks may contribute to core features of ASD, though it is unlikely to represent the primary causal mechanism. The predictive coding framework proposes that the brain interprets the world by continuously generating and updating internal models that anticipate incoming sensory signals (Gabhart et al., 2025). The synaptic and signaling abnormalities identified in genetic and systems-level studies are therefore conceptualized as disruptions of this predictive machinery, and are likely to contribute directly to core social and communicative characteristics of ASD.

Perturbations in synaptic pathways can propagate during development to alter transcriptional programs and circuit maturation, contributing to broader cognitive impairments such as deficits in social perception and predictive processing in ASD (Lybrand et al., 2021; Fan et al., 2023; Keysers et al., 2024). Non-syndromic ASD is thought to involve relatively circumscribed abnormalities in synaptic and circuit-level implementations of prediction, which manifest as subtle but pervasive problems in anticipating and interpreting social cues. Syndromic ASD is more likely to reflect broader disruptions in the formation and updating of internal models due to early regulatory perturbations in neurodevelopmental regulatory processes, leading to a more global impairment of predictive processing across cognitive domains. The distinction between syndromic and non-syndromic ASD does not map onto discrete genes or pathways; instead, it reflects varying contributions of synaptic effector pathways and developmental regulatory pathways along a shared mechanistic continuum.

### 3.2 Key signaling pathways for syndromic ASD

#### 3.2.1 Neural development and synaptic function pathways

In syndromic ASD, pathways governing dendritic spine morphogenesis, telencephalon development, and neurogenesis regulation are known to orchestrate the macrostructural assembly of the nervous system, establishing cortical areal identity, laminar organization, and long-range connectivity, with their disruption leading to widespread spine architectural abnormalities, cortical dyslamination, and imbalances in excitatory and inhibitory neuron populations (Carroll et al., 2021; Wang X. et al., 2025). These developmental abnormalities converge on cortical and limbic systems supporting social cognition, communication, and emotional regulation. Such convergence may contribute to the characteristic phenotype of syndromic ASD. Pathways governing neural development and dendritic spine morphogenesis, mediated by Rho GTPases and actin regulators, function as developmental patterning programs. Their disruption leads to widespread alterations in spine architecture and synaptic capacity across neuronal populations in ASD (Duncan et al., 2021; Jung and Park, 2022). In syndromic ASD, the observed synaptic dysfunction is conceptualized as a secondary consequence of these primary, large-scale failures in developmental construction.

Telencephalon development pathways orchestrate the formation of forebrain structures, including the cerebral cortex, hippocampus, and amygdala (Micali et al., 2023). Their integration of morphogen signaling and transcriptional programs contributes to areal specification, cortical lamination, and long-range connectivity, and their disruption may provide a developmental substrate for the distributed cognitive and emotional abnormalities observed in some syndromic forms of ASD (Ezan et al., 2021).

In parallel, neurogenesis regulation pathways impose temporal control over neural progenitor behavior. By modulating the timing of progenitor proliferation, differentiation, and cell cycle exit, these programs ensure the generation of appropriate numbers and types of neurons and glia, as well as their correct allocation to distinct regions and layers (Ciceri et al., 2024). Abnormal regulation of these processes may contribute to altered excitatory-inhibitory balance, atypical cortical lamination, and changes in network composition in ASD, although the direction and magnitude of these effects are likely to vary according to the affected gene, cell type, brain region, and developmental stage (Parker et al., 2026).

#### 3.2.2 Synaptic plasticity-related pathways

Metabolic state, neuromodulatory input, and activity-dependent changes in gene expression and protein turnover collectively modulate the capacity of synapses to undergo experience-dependent strengthening or weakening and may influence systems-level mechanisms that support adaptive behavior in ASD (Payeur et al., 2021; Luciana and Collins, 2022; Lagerlof et al., 2026). Dysregulation of these processes may contribute to atypical sensory processing, attentional regulation, emotional control, and executive functioning, particularly in some syndromic forms of ASD. Regulation of synaptic plasticity may serve as an interface between molecular synaptic mechanisms and distributed neural systems involved in adaptive behavior (Ford et al., 2022).

The proper development and function of synaptic plasticity and cognition-related pathways depend on the coordinated regulation of neuronal morphogenesis, synapse formation, and gene expression. Dendritic growth, dendritic spine development, and the regulation of synaptic structure and activity establish the cellular substrate required for experience-dependent synaptic modification (Perera-Murcia et al., 2026). In parallel, chromatin remodeling, transcriptional regulation, and epigenetic mechanisms regulate the activity-dependent gene expression necessary for long-term synaptic adaptation (Huang et al., 2021). In syndromic ASD, recurrent disruptions of genes within these higher-order tiers result in coordinated abnormalities in dendritic and spine morphology, synaptic assembly and spatial organization, and the thresholds and gain of plastic responses. These disruptions form a mechanistic link between early neurodevelopmental perturbations and widespread synaptic pathology, E/I imbalance, and higher-order cognitive and behavioral deficits.

#### 3.2.3 Signal transduction pathways

*NF1*, *BRAF*, *TSC1*, and *MTOR* encode core components of the mTOR and Ras-MAPK signaling pathways, and their disruption underscores the pivotal roles of these cascades in syndromic ASD. The mTOR pathway integrates nutrient availability, energy status, and growth factor signals to regulate cell growth, metabolism, and autophagy (Battaglioni et al., 2022), while the Ras-MAPK cascades govern proliferation, differentiation, and survival, thereby influencing cell fate decisions across multiple developmental stages (Bahar et al., 2023). In the developing nervous system, dysregulation of these signaling pathways leads to significant alterations, including abnormal expansion of brain volume, disrupted cortical lamination, and imbalanced generation of neuronal and glial populations, consistent with the macrostructural and multisystem involvement characteristic of syndromic forms. Since mTOR and Ras-MAPK activities shape the physiological states and developmental trajectories of entire cells and tissues rather than isolated synapses, pathogenic variants in these pathways exert widespread effects on brain architecture, network organization, and resultant synaptic function.

#### 3.2.4 Epigenetic regulation pathways

Epigenetic dysregulation is a prominent feature distinguishing syndromic from non-syndromic ASD. Pathways that involve the negative regulation of gene expression, along with epigenetic modifications such as DNA methylation, integrate these mechanisms to exert higher-order control over developmental transcriptional programs (Tiane et al., 2021). Disruption of genes encoding histone modifiers, chromatin remodelers, or associated complexes leads to perturbations that extend beyond individual targets, resulting in widespread disorganization of transcriptional programs essential for neurogenesis, regional patterning, and synaptic differentiation (Mossink et al., 2021; Jiang et al., 2023). This global dysregulation offers a coherent explanation for the broad neurodevelopmental impairments and frequent multisystem involvement characteristic of syndromic ASD. It highlights epigenetic and chromatin organization pathways as central axis that differentiates syndromic from non-syndromic genetic architectures. Emerging evidence underscores the critical role of chromatin modifiers in orchestrating neurodevelopmental programs, with dysregulation linked to ASD pathogenesis (Rezazadeh et al., 2025).

#### 3.2.5 Circadian rhythm pathway

The circadian timing system, through transcriptional feedback loops involving clock genes such as *CLOCK*, *BMAL1*, *PER*, and *CRY*, regulates the rhythmic expression of genes critical for neurodevelopment and synaptic function. Disruption of this system is a common comorbidity in ASD and is implicated in exacerbating sleep disturbances, behavioral rhythm abnormalities, and emotional dysregulation, potentially by dysregulating the temporal coordination of neuronal and synaptic processes (Abe et al., 2022; Hoyt and Obrietan, 2022; Lane et al., 2023). These processes are particularly crucial during key developmental periods when precise temporal gene expression is essential for proper circuit formation. Disruptions in circadian and ultradian rhythmic regulation may contribute to the high prevalence of sleep disturbances, behavioral rhythm abnormalities, and emotional instability observed in syndromic ASD. Furthermore, such disruptions may interfere with the timing of developmental programs that shape neuronal connectivity and function.

#### 3.2.6 Morphogenesis and growth pathways

Pathogenic variants in morphogenesis and growth-related pathways frequently influence programs governing multi-organ development rather than exclusively targeting brain circuits (St Peter et al., 2024). The regulatory systems underlying developmental growth and positive regulation of growth coordinate the size, shape, and proportional development of the body and craniofacial structures across both embryonic and postnatal stages (Zhao et al., 2026). These pathways integrate proliferative cues, patterning signals, and tissue-specific growth factors to ensure synchronized and anatomically coherent development of skeletal, craniofacial, visceral, and neural structures (Wang et al., 2026). Disruption of these morphogenesis and growth pathways, which govern the development of multiple organ systems, leads to multisystem involvement and distinctive somatic features characteristic of syndromic ASD (Palma-Milla et al., 2024). The neurodevelopmental and behavioral manifestations of these syndromes converge in the nervous system, where altered cell proliferation, migration, and differentiation disrupt synaptic assembly and circuit formation. Thus, the phenotypic severity of syndromic ASD reflects both the broad impact of morphogenetic pathway dysregulation and its final common pathway of synaptic dysfunction.

Comparative overview of key signaling pathways enriched in non-syndromic and syndromic ASD is presented in Table 2.

**Table 2:** Comparative overview of key signaling pathways enriched in non-syndromic and syndromic autism spectrum disorder.

Pathway category	Non-syndromic ASD		Syndromic ASD	
	Pathway/Biological process	Key features and ASD relevance	Pathway/Biological process	Key features and ASD relevance
Synaptic assembly and adhesion	Neuroligin–Neurexin–Shank scaffold pathway	Mediates trans-synaptic pairing and excitatory synapse maturation; rare variants in *NLGN3*/*4X*, *NRXN1*–*3*, and *SHANK2*/*3* directly disrupt synapse number and geometry, producing core ASD phenotypes without systemic anomalies.	Dendritic spine morphogenesis pathway	Rho-GTPase and actin-regulatory machinery controls spine number, size, and shape across large neuronal populations; disruption causes widespread spine architectural abnormalities and broad neurodevelopmental impairment.
Excitatory neurotransmission	Glutamatergic synapse pathway (NMDA/AMPA receptor trafficking and signaling)	Supports LTP, synaptic plasticity, and information encoding in cortical and hippocampal networks; variants in *GRIN1/2A/2B* and *GRIA2* alter receptor kinetics and calcium signaling.	Telencephalon development pathway	Integrates morphogen gradients and transcription factor cascades to establish cortical areal identity, laminar organization, and long-range connectivity; perturbation affects multiple cognitive and emotional systems simultaneously.
Neuronal excitability and E/I balance	Voltage-gated Na^+^/K^+^ channel and GABA type A receptor pathways	*SCN2A/8A*, *KCNQ2/3*, and *GABRB2/3* regulate action potential firing, membrane stability, and inhibitory tone; dysfunction shifts E/I balance at the circuit level.	Neurogenesis regulation pathway	Controls timing of progenitor proliferation, differentiation, and cell-cycle exit; dysregulation causes E/I neuron imbalances, cortical dyslamination, and altered network composition.
Synaptic plasticity	Ras/Rap1–LTP cascade (activity-dependent plasticity)	NMDA receptor activation → Ca^2+^ influx → kinase cascades → AMPA receptor insertion; Ras and Rap1 translate synaptic activity into localized changes in receptor trafficking, cytoskeletal remodeling, and spine stability.	Higher-order plasticity regulation (metabolic, neuromodulatory, transcriptional control)	Encompasses metabolic state, neuromodulatory input, and activity-dependent gene expression/protein turnover; depends on upstream chromatin remodeling and developmental programs for long-term synaptic change.
Intracellular signal transduction	Ras–MAPK and Rap1 pathways (local, synapse-restricted)	Couple extracellular cues to localized synaptic responses; modulate receptor localization, protein synthesis, and cytoskeletal organization within single neurons or microcircuits.	mTOR and Ras–MAPK cascades (global, cell-wide)	mTOR integrates nutrient, energy, and growth factor signals to regulate cell growth, metabolism, and autophagy; *TSC1*, *MTOR*, *PTEN*, *NF1*, *BRAF* mutations produce macrostructural brain changes and multisystem involvement.
Epigenetic and transcriptional regulation	—	Non-syndromic ASD pathways are often enriched at the effector level, although upstream regulatory influences may also contribute.	Chromatin remodeling and gene expression regulation	*CHD7*, *SETD5*, *KMT2A*, *CREBBP*, *MEF2C*, *ADNP* modify histone marks, TAD organization, and spatiotemporal transcription programs; disruption causes genome-wide misregulation and multisystem developmental anomalies.
Protein homeostasis	Local translational control at synapses (FMRP-mediated)	*FMR1*/FMRP regulates local mRNA translation at synapses; alterations affect synaptic proteome composition and excitatory network homeostasis.	Ubiquitin–proteasome pathway (global)	*UBE3A* (E3 ligase) and *USP9X* (deubiquitinase) control substrate protein half-life and abundance; imbalance disrupts synaptic number, structure, and plasticity at a systemic level.
Circadian and rhythmic regulation	—	Circadian disruption is a common comorbidity in ASD and may contribute to sleep disturbances, behavioral rhythm abnormalities, and emotional dysregulation.	Circadian timing pathway	*CLOCK*, *BMAL1*, *PER*, *CRY* feedback loops control sleep–wake, endocrine, and metabolic rhythms; disruption contributes to sleep disturbances, behavioral rhythm abnormalities, and emotional instability.
Morphogenesis and growth	—	Non-syndromic ASD often shows predominantly circuit- and synapse-level dysfunction, with macrostructural or somatic abnormalities occurring less consistently.	Morphogenesis and growth regulation	Pathways governing multi-organ development (craniofacial, skeletal, visceral) produce the distinctive somatic features of syndromic ASD; altered cell proliferation/migration ultimately converges on synaptic assembly defects.

An em dash (—) indicates pathways that are not prominently enriched in that ASD subtype. ASD, autism spectrum disorder; AMPA, α-amino-3-hydroxy-5-methyl-4-isoxazolepropionic acid; E/I, excitation/inhibition; FMRP, fragile X messenger ribonucleoprotein; GABA, γ-aminobutyric acid; LTP, long-term potentiation; MAPK, mitogen-activated protein kinase; mTOR, mammalian target of rapamycin; NMDA, N-methyl-D-aspartate; TAD, topological associating domain.

### 3.3 A dimensional hierarchy of biological pathways

The distinctions between non-syndromic and syndromic ASD may reflect a continuum in the molecular scope and developmental consequences of pathogenic variation, ranging from alterations in synaptic proteins and neuronal excitability to abnormalities in chromatin remodeling, transcriptional regulation, intracellular signaling, and neuronal development (Sztainberg and Zoghbi, 2016). This continuum is exemplified by key genes and pathways, including those involving *SHANK3*, *FMR1*, *UBE3A*, and mTOR. In syndromic forms, mutations in genes such as *TSC1*, *TSC2*, or *PTEN* lead to constitutive activation of mTOR signaling, driving aberrant cellular growth and structural brain anomalies (Aronica et al., 2023; Karalis et al., 2024). In non-syndromic ASD, alterations in mTOR signaling may preferentially affect synaptic protein translation, dendritic development, and activity-dependent plasticity without necessarily producing macroscopic brain abnormalities. Similarly, haploinsufficiency of *SHANK3* is commonly associated with Phelan-McDermid syndrome and broad developmental impairments, whereas specific missense variants may produce more restricted or clinically variable synaptic phenotypes (Varella-Branco et al., 2026). Alterations in *FMR1* and *UBE3A* are most strongly associated with syndromic neurodevelopmental disorders, although differences in variant type, gene dosage, molecular mechanism, and tissue specificity can influence the resulting phenotype.

The pathway-level distinctions between non-syndromic and syndromic ASD reveal a dimensional hierarchy of biological disruption. Comparative overview of signaling pathway characteristics is presented in Table 3.

**Table 3:** Comparison of overall characteristics of signaling pathways between non-syndromic ASD and syndromic ASD.

Dimension for comparison	Non-syndromic ASD	Syndromic ASD
Core focus	Real-time functional expression for regulating synaptic communication and neuronal signal processing	The procedural blueprint and spatiotemporal progression governing neural development
Path property	Functional pathway: Directly mediates intercellular communication, signal transduction, and electrophysiological activities	Regulatory pathways: Control of developmental timing, cell fate determination, and large-scale morphogenesis
Heterogeneity	The pathways are relatively concentrated, focusing on synapses, adhesion, and signal transduction.	The pathways exhibit extensive coverage, spanning from chromatin to morphogenesis, with a rich hierarchical structure.

ASD, autism spectrum disorder.

## 4 Discussion

This review provides a comparative overview of syndromic and non-syndromic forms of ASD, arguing that its apparent genetic heterogeneity follows orderly biological hierarchies rather than being random or idiosyncratic.

### 4.1 Continuum framework for ASD pathogenesis

This review advances a three-layer hierarchical framework that unifies molecular genetics with systemic pathophysiology to interpret ASD etiology, as presented in Figure 1. The model organizes gene function (Layer 1), core biological pathways (Layer 2), and clinical phenotypes (Layer 3) into a three-tiered hierarchy, conceptualizing syndromic and non-syndromic ASD as a biological continuum from local synaptic alterations to broader circuit and systemic dysregulation.

**Figure 1:**
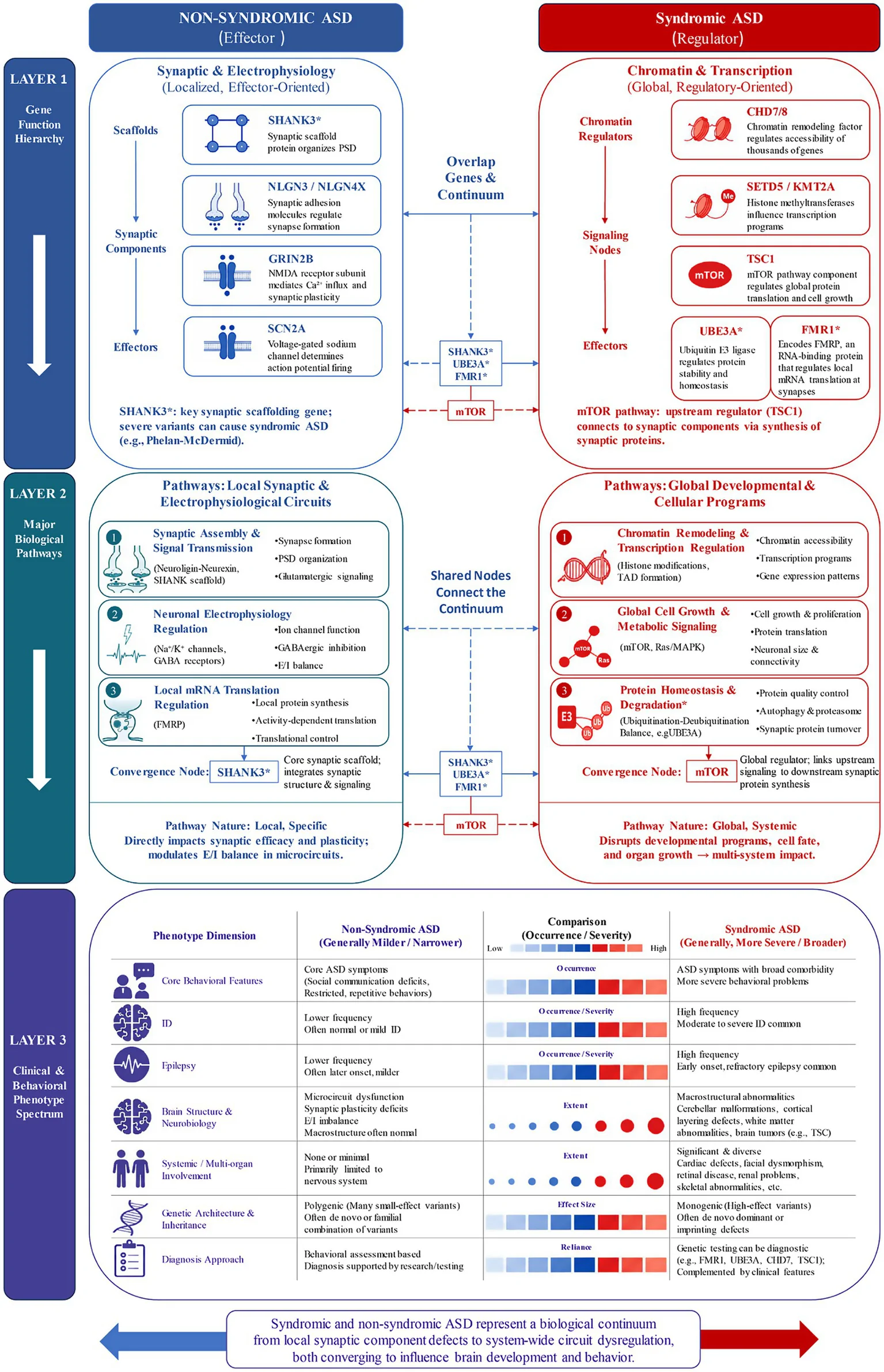
A three-layer hierarchical framework from genotype to phenotype in syndromic and non-syndromic ASD. Schematic framework stratifying ASD genetic and molecular architecture into three hierarchical layers linking gene function (Layer 1), core biological pathways (Layer 2), and clinical phenotypes (Layer 3). Genes and pathways are mapped onto a functional continuum from local synaptic effectors to global regulatory programs, with dashed lines denoting mechanistic convergence nodes (e.g., SHANK3, mTOR). The asterisk (*) indicates overlapping genes with dual relevance across both ASD subtypes. ASD, autism spectrum disorder; E/I, excitation/inhibition; FMRP, fragile X messenger ribonucleoprotein; GABA, γ-aminobutyric acid; ID, intellectual disability; MAPK, mitogen-activated protein kinase; mTOR, mammalian target of rapamycin; NMDA, N-methyl-D-aspartate; PSD, postsynaptic density; TAD, topological associating domain.

Distinctive features of this framework. Unlike conventional gene–pathway–phenotype models that may treat risk genes as functionally equivalent nodes, the proposed hierarchy distinguishes three functional categories according to the breadth of their biological effects. Local effectors (e.g., *SHANK3*, *GRIN2B*) primarily influence specific synaptic and cellular functions, whereas global regulators (e.g., *CHD7*, *TSC1*) affect broader chromatin, transcriptional, or cellular growth programs. Bridge genes (e.g., *SHANK3*, *FMR1*, *UBE3A*) occupy an intermediate position by linking regulatory processes with downstream cellular and synaptic functions. These categories are conceptual rather than mutually exclusive, and their effects may vary with variant type, dosage, developmental timing, and cellular context. This stratification generates testable predictions: broader regulatory perturbations may be associated with more widespread developmental phenotypes, whereas local effector perturbations may show more synapse- or circuit-proximal effects, with bridge genes showing intermediate or context-dependent phenotypic spectra. It also suggests that therapeutic responses may differ according to the affected biological tier.

The framework further illustrates how pathogenic variation can produce heterogeneous phenotypic outcomes depending on variant severity, dosage sensitivity, developmental timing, genetic background, and environmental context. Conversely, alterations in different genes or pathways may converge on shared biological processes and behavioral features. This may help explain why individuals with different genetic findings can exhibit overlapping behavioral phenotypes, while individuals with alterations in the same gene may differ in cognition, epilepsy risk, sensory processing, and systemic involvement.

Variants affecting chromatin regulation or broad cellular signaling may be associated with transcriptomic, epigenomic, proteomic, metabolic, or neurodevelopmental biomarkers, highlighting potential opportunities for biomarker development.

At the therapeutic level, alterations in ion channels, neurotransmitter receptors, or synaptic signaling may be amenable to interventions targeting neuronal excitability or receptor function, whereas dysregulation of mTOR signaling, protein synthesis, or protein degradation may require pathway-directed approaches with broader cellular effects. Chromatin and transcriptional disorders may require interventions aimed at restoring specific expression programs within defined developmental windows. Shared biological convergence points could further support treatments across multiple genetic conditions, while the framework may help guide treatment selection according to disease mechanism and developmental timing.

### 4.2 Limitations

Current evidence is further limited by unequal gene discovery, small cohorts for rare variants, ascertainment bias toward individuals with marked developmental impairment, and limited representation across age, sex, ancestry, and clinical presentation. Many proposed links between genes, pathways, biomarkers, and behavior are based on model systems and remain incompletely validated in human neural tissue.

### 4.3 Future directions

Future research on ASD could utilize cohort designs that integrate hierarchical gene functions with diverse clinical phenotypes. It is essential to reevaluate the distinctions between syndromic and non-syndromic ASD through comprehensive analyses of molecular, synaptic, circuit, and structural alterations. Combining genomic, transcriptomic, neuroimaging, electrophysiological, and behavioral measures may clarify the relationships among genetic variation, molecular pathways, neural alterations, and behavioral phenotypes. Validating reproducible biomarkers and genetic findings in representative clinical populations may further advance biological stratification, prognostic assessment, and personalized intervention. A strategic map for future research on ASD is presented in Figure 2.

**Figure 2:**
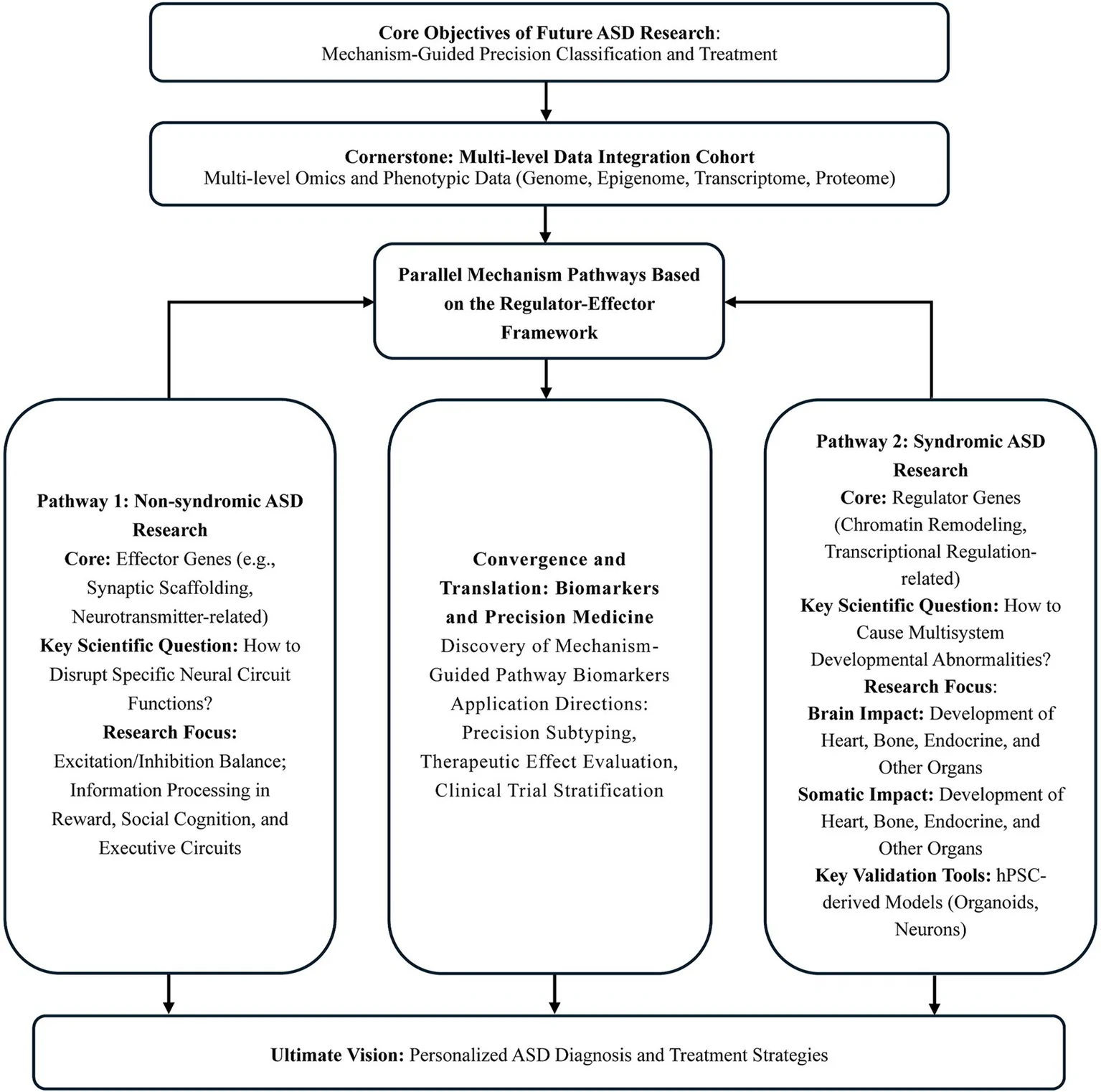
Strategic map for future research on autism spectrum disorder. ASD, autism spectrum disorder; hPSC, human pluripotent stem cell.

## Footnotes

1
https://gene.sfari.org/
